# Supplementary figures and images for: Honey Bee Parasitic Mite Contains the Sensilla-Rich Sensory Organ on the Foreleg Tarsus Expressing Ionotropic Receptors With Conserved Functions
Source: Front Physiol. 2019 May 9;10:556. doi: 10.3389/fphys.2019.00556 (PMC6520597; doi:10.3389/fphys.2019.00556)

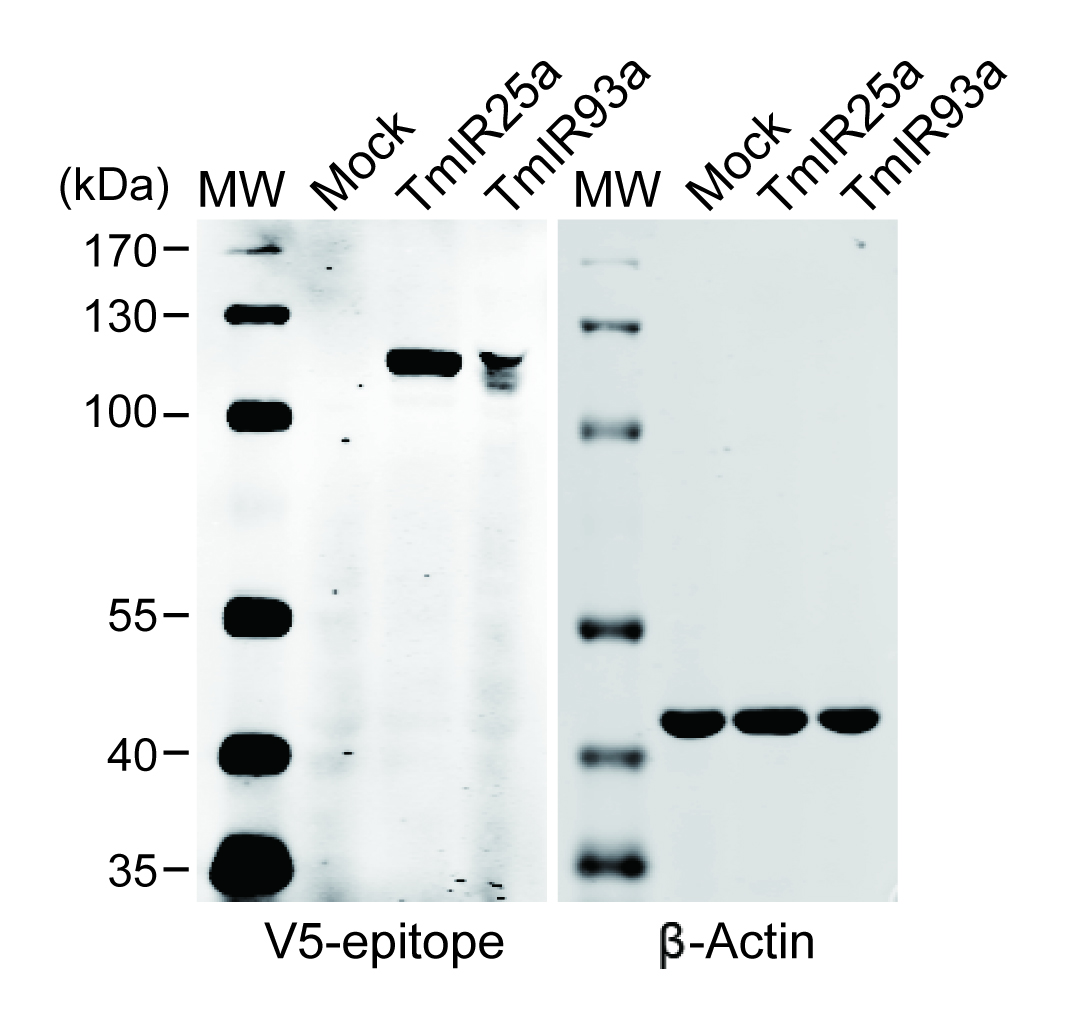

Supplement: FIGURE S1 — Expression of TmIR25a and TmIR93a proteins. The IR proteins (V5-epitope) and β-actin expressed in HEK293 cells transfected with empty vector (Mock), TmIR25a-, and TmIR93a-expressing constructs were analyzed by western blot. The size (kDa) of protein molecular weight marker (MW) is at the left. [file Image_1.JPEG]
